# The Advances and Challenges of Liposome-Assisted Drug Release in the Presence of Serum Albumin Molecules: The Influence of Surrounding pH

**DOI:** 10.3390/ma15041586

**Published:** 2022-02-20

**Authors:** Danuta Pentak, Anna Ploch-Jankowska, Andrzej Zięba, Violetta Kozik

**Affiliations:** 1 Faculty of Chemistry, University of Opole, Oleska 48, 45-052 Opole, Poland; aniaploch@op.pl; 2 Department of Organic Chemistry, Faculty of Pharmaceutical Sciences in Sosnowiec, Medical University of Silesia in Katowice, Jagiellońska 4, 41-200 Sosnowiec, Poland; zieba@sum.edu.pl; 3 Institute of Chemistry, University of Silesia, Szkolna 9, 40-006 Katowice, Poland

**Keywords:** biological systems, release mechanism, controlled drug delivery, nanoparticles

## Abstract

The aim of this study is to prepare a liposomal delivery system for 5-methyl-12 (H)-quino[3,4-b]-1,4-benzothiazine chloride (5-MBT) and study the in vitro release characteristics. The release of 5-MBT from a liposomal complex with human serum albumin (HSA) [L_DPPC/5-MBT_]:HSA was examined using the spectrophotometric method and differential scanning calorimetry (DSC). Electronic paramagnetic resonance was used to assess the influence of the pH of the environment on the conformation of phospholipids, the latter determining the degree of release of the encapsulated compound. The applied mathematical models made it possible to determine the necessary analytical parameters to facilitate the process of potential drug release from liposomes. The complexes formed by liposomal 5-MBT with serum albumin (HSA) particles allowed for the description of the Fick process. The change in the polarity of the phospholipid membrane resulting from the changes in the pH of the surroundings, significantly influenced the percentage of 5-MBT entrapment in the liposomes. It also affected the release percentage.

## 1. Introduction

Nanotechnology is among the most dynamically developing fields of science and technology. It is an interdisciplinary field that combines elements of modern chemistry, physics, biology and computer science. It plays an increasingly important role in various areas of life, especially in pharmacy and medicine [1].

Recent years have witnessed an intensively increased interest in drug delivery systems for targeted therapy based on the use of nanoparticles. Therapeutic approaches of this type play an important role in the treatment of specific cancers [2], diabetes [3], viral and fungal infections [4], and also increasingly in gene therapy [5]. Nanoparticles as carriers of active substances facilitate drug transport to specific sites in the body, improving the pharmacodynamic and pharmacokinetic properties of the drug (the increased bioavailability and release time of the active substance, along with extended duration of the pharmacological action of the administered drug, and reduced toxicity). The solubility and stability of the active substance can also be improved [6].

One of the most common nano-scale carriers for drug transport are liposomes [7,8,9]. Intensive research has been carried out for many decades aimed at developing new techniques for forming liposomes for the targeted delivery of drugs and other substances to specific sites in the biological system.

Liposomes are artificially produced spherical vesicles consisting of a lipid bilayer enclosing an aqueous solution [10]. Due to their properties, liposomes can be carriers of anti-cancer drugs [11]. The degree of drug encapsulation in liposomes depends on the method of liposome preparation. The thermodynamic state of the liposomal membrane, i.e., the mobility of the molecules that form it, depends mainly on the temperature. Phospholipids are characterized by a phase transition temperature (Tc) above which the lipid bilayer is present in the liquid crystalline phase, whereas it is present below in the gel phase. The liposomal membrane in the gel phase is stiff and orderly with limited mobility of the molecules, while in the liquid phase (liquid crystalline) it is disordered and leaky. The liposomal membrane phase determines the permeability, aggregation, protein binding and, to a lesser extent, liposome fusion. As the phase transition temperature varies with the length and type of (saturated or unsaturated) fatty acid chains, the fluidity of the bilayer can be controlled by selecting different phospholipids. The Tc of phospholipids ranges from −20 °C to 90 °C [12].

For some applications, the permeability of liposomal membrane is a problem because of the premature leakage of the liposome-encapsulated substance, reducing its therapeutic effectiveness at the targeted site.

From the perspective of using liposomes as carriers of therapeutic substances, the temperature of the target site and the surrounding pH should be taken into account during design [13].

The results of pharmaceutical availability studies conducted under laboratory conditions (in vitro) do not always reflect the in vivo behavior of the drug, e.g., when present in the human body. However, they are very important in determining drug quality and predicting its therapeutic efficacy. In most cases, these studies do not take into account the influence of various factors present in the body upon the release and absorption of the drug. These factors include the pH of the environment.

The use of liposome-encapsulated drugs makes it possible to reduce the dose of active ingredient and the frequency of administration, thereby reducing the overall toxicity of the drug, while ensuring the desired therapeutic effect. Drugs delivered to the body in a liposomal form stay longer in the bloodstream, so their pharmacokinetics and biodistribution are improved.

The possibility of systemic delivery of anti-cancer substances using liposomes as carriers is due to the fact that endothelium of tumor blood vessels is not as tight as the endothelium in healthy tissues, and this facilitates penetration of the drug-containing liposomes into the surrounding tissues [14]. The size of the intercellular spaces in the tumor capillary endothelium is 100–780 nm depending on the type of tumor, while in the normal endothelium these spaces do not exceed 5–10 nm [15]. Moreover, solid tumors lack adequate lymphatic drainage, which results in the accumulation of liposomes in the tumor tissue. This effect, called the Enhanced Permeability and Retention (EPR) effect, is used as an effective method of passively targeting carriers, such as liposomes, to the tumor site [16]. Low molecular weight drugs, unlike liposomes, are not able to accumulate in the tumor for a long time and are returned to the bloodstream very quickly. An important parameter influencing passive steering by the mentioned effect is the diameter of the carriers. It is assumed that liposomes designed for in vivo use should be less than 400 nm in diameter. On the other hand, research shows that liposomes with a diameter of less than 200 nm reach the tumor microenvironment most effectively [17].

**Figure 1:**
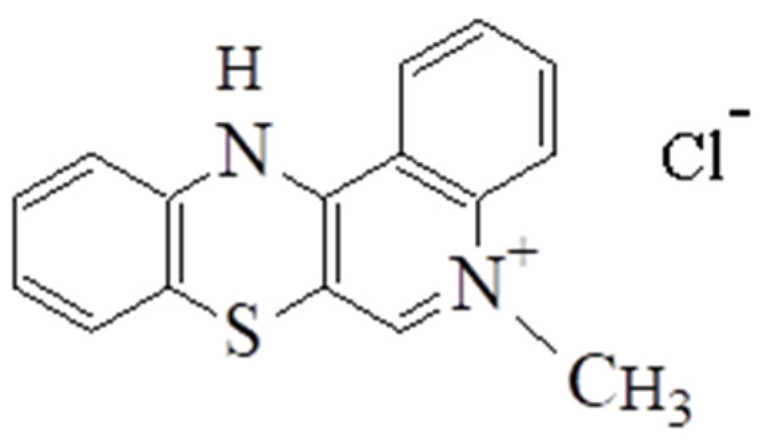
Chloride-5-methyl-12(H)-chino[3,4-b]-1,4-benzothiazine.

The aim of the study is to encapsulate into liposomes a compound with potential anticancer activity, i.e., 5-methyl-12(H)-quino[3,4-b] [1,4] benzothiazine (5-MBT) chloride (Figure 1).

5-Alkyl-12(H)-quino[3,4-b] [1,4] benzothiazine salts exhibit antiproliferative properties. The analyzed compound was tested in vitro for antitumor activity using two cultured cell lines: HCT 116 human colon cancer (American Type Culture Collection, Rockville, MD, USA) and lung cancer LLC (Institute of Immunology and Experimental Therapy, Wrocław, Poland) using doxorubicin as a reference. The tested compound showed activity against both tumor cell lines [18].

One of the mechanisms involved in the antiproliferative activity of chemotherapeutic agents is DNA intercalation. This mode of action is typical of antiproliferative anthracycline antibiotics, e.g., doxorubicin. The 5-alkyl-12(H)-quino[3,4-b] [1,4] benzothiazine salts have a similar structure, thus it can be assumed that these compounds possess similar antiproliferative mechanism of action. The research has shown that the 1,4-thiazine ring is responsible for antiproliferative activity of 5-alkyl-12(H)-quino[3,4-b] [1,4] benzothiazine salts. The compounds with substituents in the 9 and 10 position of the quinobenzothiazine ring and 5-methyl-12(H)-chloride have the highest antitumor activity among the 5-alkyl-12(H)-quino[3,4-b] [1,4] benzothiazines, the latter without additional substituents [18]. Obtaining the liposomal form of 5-methyl-12(H)-quino[3,4-b] [1,4] benzothiazinium chloride (5-MBT) allowed us to analyze the influence of the surrounding pH on the process of 5-MBT release from liposomes.

In order to analyze more precisely the process of 5-MBT release from liposomes under conditions simulating an in vivo situation, the tested samples were exposed to human serum albumin (HSA). The tests were carried out at 37 °C.

## 2. Materials and Methods

### 2.1. Materials

L-α-phosphatidylcholine dipalmitoyl (1,2-dihexadecanoyl-*sn*-glycerol-3-phosphocholine) (DPPC, purity 99%), chloroform, dichloromethane, hydrochloric acid, acetic acid 80%, acetic acid sodium salt trihydrate, disodium hydrogen phosphate, dipotassium hydrogen phosphate, 2-(3carboxypropyl)-4,4-dimethyl-2-tridecyl-3-oxazolidinyloxyl (5-DOXYL), and α,α,α–Tris-(hydroxymethyl)-methylamin (TRIS, purity 99.9%) were purchased from Sigma-Aldrich, Schnelldorf, Germany. Human serum albumin (HSA) fraction V, crystallized and lyophilized, was purchased from MP Biomedicals, France. Chloride-5-methyl-12(H)-quino[3,4-b]-1,4-benzothiazine (5-MBT) was synthesized at the Department of Organic Chemistry, Medical University of Silesia in Katowice.

### 2.2. Methods

#### 2.2.1. The Synthesis of 5-Methyl-12(H)-quino[3,4-b] [1,4] Benzothiazine Chloride

12 (H)-quino[3,4-b] [1,4] benzothiazine salts are formed by the reaction of 5,12- (dimethyl) thiquinanthrenedium bischloride with aromatic amines. The structure of the reaction products depends on the presence of oxygen in the reaction mixture. If the reaction of the bis-chloride with aromatic amines is carried out in the presence of atmospheric oxygen with vigorous stirring, the reaction products are the corresponding 12 (H)-quino[3,4-b] [1,4] benzothiazine salts. When the reaction is carried out in the absence of atmospheric oxygen, the reaction gives 1-alkyl-4- (arylamino) quinoline-3-thiolanes. In the presence of aniline hydrochloride and atmospheric oxygen, the 3-thiolates are cyclized to the corresponding quinobenzothiazine derivatives. In this case, the 3-thiolates act as intermediates in the reactions of bis-salts with aromatic amines in the presence of oxygen, and these reactions lead to the formation of the quinobenzothiazine salt.

The course of reaction between 5,12-(dimethyl) thiquinanthrenodium bis-chloride and 2-fluoroaniline and 2-choloroaniline is not dependent on the presence of oxygen in the reaction mixture. The 3-thiolates formed at the first stage of the reaction are cyclized to 1,4-thiazine not by substitution with a hydrogen atom, but by substituting a halogen atom with sulfur. In nucleophilic substitution reactions, the fluoride and chloride anions are easy leaving groups and do not require the presence of an oxidizing agent. Such reactions lead to a product having the structure of 5-methyl-12(H)-quino[3,4-b] [1,4] benzothiazine chloride [18] (Figure 1).

#### 2.2.2. Liposome Preparation

The liposomes were prepared by modified reverse-phase evaporation method (mREV), which relies on the continuous mixing of specific volumes of suitable phospholipid solutions in excess of organic solvents with water phase. The REV method was described by Papahadjopoulos [19]. The modification of this method consisted of replacing the ultrasonic dispersion of the aqueous phase in the organic phase with mechanical dispersion that allows complete conversion of the phospholipids to the liposomes [20].

Liposomes (L_DPPC_; L_DPPC/5-MBT_) were obtained by the mREV method using the DPPC:drug molar ratio 30:1. The lipid dispersion at a final lipid concentration of ca. 2.64 × 10^−2^ M was used. A total of 0.34 mL of 5-MBT at a concentration of 5 × 10^−3^ M was added to the preparation mixture. Next, a buffer (2 mL) with an appropriate pH (2.00; 4.47; 5.50; 5.59; 6.10; 7.40; 8.00; 8.40) and 4 mL of organic solution prepared from methylene chloride and chloroform (3:1) were applied. For the electron paramagnetic resonance (EPR) study, 0.016 mL of the spin marker 5-DOXYL (12.5 mg/mL in chloroform) was added to the preparation mixture. The preparation process was carried out at the temperature of 44 °C. The average time of liposome preparation did not exceed 12 min. The liposome-entrapped analyzed drug was separated from the free drug by dialysis (at 4 °C) using Float-A-Lyzer G2 (Spectra/Por) tubing and several changes of buffer.

#### 2.2.3. Solutions and Sample Preparation

Acetate buffer pH 4.47, phosphate-buffered saline (PBS) and pH 5.59 and 6.10 were used for the UV/Vis and NMR measurements. Deuterated solvents were used for NMR studies. For the EPR experiment, Tris-HCl buffers (pH 2.0; 5.5; 7.4; 8.0 and 8.4) were prepared.

For the human serum albumin-drug interaction analysis, a stock solution of 5-MBT in H_2_O (5.0 × 10^−3^ M) was prepared. Stock solutions of human serum albumin (HSA) at the concentration of 1.0 × 10^−3^ M were prepared with appropriate apH. All mixtures used in the reactions were prepared in triplicate to allow for statistical analysis.

#### 2.2.4. UV/Vis Measurements

The absorption spectra of free 5-MBT, the liposomal form of 5-MBT, and of the liposome-drug-albumin systems were recorded with the Lambda Bio 40 spectrometer (Perkin Elmer, Walthman, MA, USA) equipped with a PTP-1 Peltier System (Perkin Elmer, Walthman, MA, USA) automatic temperature controller. The temperature was maintained at 37 ± 0.1 °C. All the spectra were acquired after equilibration of the samples with the automatic temperature controller. The spectral analysis was performed using UV WinLab Perkin Elmer Software.

##### Encapsulation Efficiency and Drug Loading

Immediately after preparing the liposomal form of 5-MBT (L_DPPC/5-MBT_), the samples were dialyzed in order to separate the encapsulated drug from its unencapsulated form. Aliquots (8 mL) of liposomal form of the analyzed drug dispersion were placed into a Float-A-Lyzer G2 (Spectra/Por) cellulose ester dialysis tubing (Spectra/Por^®^, 8–10 kilodaltons MWCO, Spectrum Laboratories, Inc., Rancho Dominguez, CA, USA), immersed in 50 mL buffer at 4 °C with magnetic stirring at 360 rpm. The samples taken from the recipient solution at predetermined times were replaced with the same volumes of fresh buffer and spectrophotometrically assayed at 241 nm for the 5-MBT content. The concentration of the encapsulated drug was analyzed using the standard curve of the drug by measuring the maximum absorption of the 5-MBT (λ_max_ 241 nm). The encapsulation efficiency (*EE*%) of 5-MBT entrapped within the liposomes was calculated from Equation (1) [21]. The drug loading (*DL*%) was calculated according to Equation (2) [22]:

(1) $$EE\%=\frac{C{\;}_{total}-C_{free}}{C_{total}}\times 100$$

(2) $$DL\%=\frac{C{\;}_{total}-C_{free}}{C_{total\;lipid}}\times 100$$

where:

*C_total_*—total concentration of the drug;

*C_free_*—concentration of the free drug in the supernatant;

*C_total lipid_*—total concentration of the lipid.

#### 2.2.5. Electron Paramagnetic Resonance

**Figure 2:**
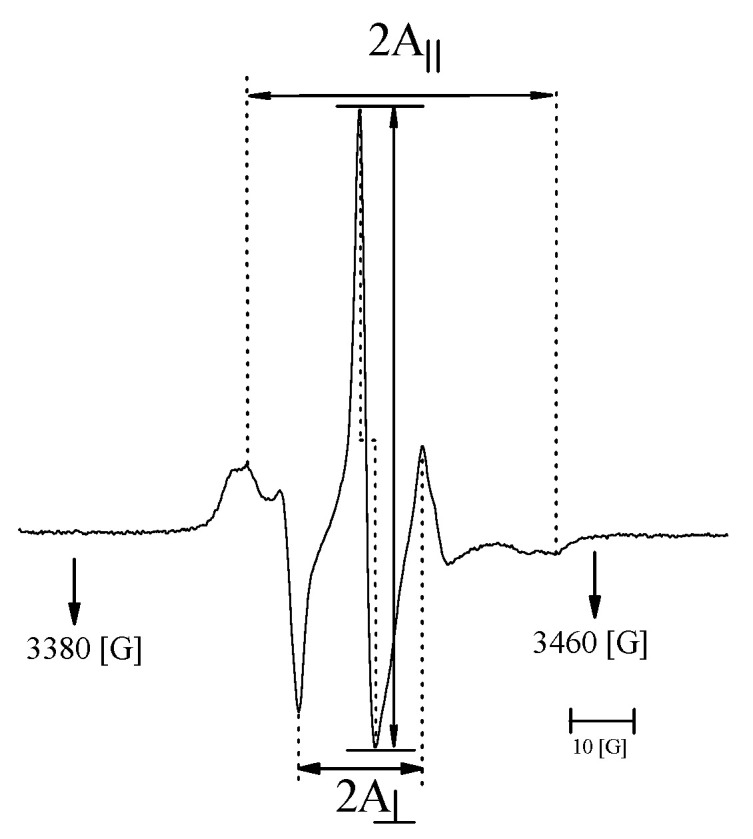
Typical EPR spectrum of spin marker 5-DOXYL located in the liposome membrane.

Electron paramagnetic resonance spectroscopy (EPR) was used to monitor the molecular dynamics of the lipids at different surrounding pH levels. The EPR measurements were carried out on Bruker EMX spectrometer (Rheinstetten, Germany) at the X-band (9 GHz), equipped with a Bruker N_2_ temperature controller in the 24–57 °C temperature range maintained within ±0.5 °C during the experiment. All the spectra were acquired after the equilibration of the samples with the automatic temperature controller. The spectra (10 scans) were recorded on microwave power 20.070 mW, with 4.48 × 10^4^ signal amplification, 0.80 G modulation amplitude, and a sweep time of 20.973 s. For the EPR experiment, 0.1 mL of liposome sample solutions was kept in closed quartz capillaries. The EPR study of liposomes labeled by the spin marker 5-DOXYL allows for us to determine the 2*A_II_* and *a*′*N* parameters (Figure 2).

The parameter *a*′*N* Equation (3) obtained on the basis of the spectra allows us to determine the polarity of liposomal membranes and their fluidity, depending on the polarity of surroundings during liposome preparation. The increase in the value of the *a*′*N* parameter indicates an increase in the polarity of the membrane [23].

(3) $$a^{\prime }N=\frac{1}{3}\left(A_{II}+2A_{⊥}\right)$$

2*A*_⊥_ and 2*A_II_* are the hyperfine constants determined with the magnetic field perpendicular and parallel to the membrane normal.

Parameter 2*A_II_* is the distance between the outer splitting maxima of the 5-DOXYL hyperfine EPR spectrum embedded in the liposomal membrane. 2*A*_⊥_ is the distance between the inner maxima. The increase in the value of the 2*A_II_* parameter is related to the decreased fluidity of the liposomal membrane [24,25].

#### 2.2.6. Drug Release and the Mathematic Modeling Study

The study of chloride-5-methyl-12(H)-quino[3,4-b]-1,4-benzothiazine (5-MBT) release from liposomes (L_DPPC/5-MBT_) and complexes with human serum albumin ([L_DPPC/5-MBT_]:HSA) was carried out at 37 °C for 7 h. The degree of drug release from liposomes and albumin complexes was determined spectrophotometrically. During the first 60 min, the measurements were carried out every 5 min. After this time, the measurements were conducted every 15 min. Drug release (*R*%) was calculated according to Equations (4) and (5) [26].

(4) $$R\left(\%\right)=\frac{\left([x{]}_{f\;}-[x{]}_{fo}\right)}{(\left[x{]}_{t}\times EE\%\right)}\times 100$$

where:

R(%)—encapsulated drug percentage of leakage;

${\left[x\right]}_{f\;}$—concentration of the leaked drug;

${\left[x\right]}_{fo}$—initial concentration of the unencapsulated drug.

(5) $${\left[x\right]}_{t\;}=\frac{{\left[x\right]}_{to}}{\beta }$$

${\left[x\right]}_{to}$—concentration of total drug in the original liposomes;

β—dilution;

EE%—percentage of drug encapsulation.

To analyze the process and degree of drug release from the liposomes, the following mathematical models were used (6–10) [27,28]:

(6) $$\mathrm{First-order}:\;X=1-e^{-k\left(t-\propto \right)}$$

(7) $$\mathrm{Higuchi}:\;X=k{\left(t-\propto \right)}^{0.5}$$

(8) $$\mathrm{Bhaskas}:\;X=1-e^{-k{\left(t-\propto \right)}^{0.65}}$$

(9) $$\mathrm{Ritger-Peppas}:\;X=k{\left(t-\propto \right)}^{n}$$

(10) $$\mathrm{Korsmeyer-Peppas}:\;X=kt^{n}$$

where:

*X*—release percentage (*R*(%) from the fitting of experimental data to the mathematical formulas);

*t*—release time;

*k*—kinetic constant;

*α*—modified parameter;

*a*—the fit factor;

*n*—exponent describing the various mechanisms of release.

The value of *n* < 0.45 corresponds to Fick’s diffusion and the amount of fractions released from the carrier is then proportional to the square root of the time value. When 0.45 < *n* < 0.89, a process other than Fick’s diffusion occurs and drug release takes place as a result of both the diffusion and controlled mechanisms [29,30,31,32,33,34,35,36]. The applied equations were evaluated using the residual sum of squares (SUM) and R^2^_adj_ values.

#### 2.2.7. Differential Scanning Calorimetry

Differential scanning calorimetry (DSC) studies of the obtained liposomal preparations were performed to determine the phase transition temperature of the phospholipids. DSC scans were performed using the VP DSC ultrasensitive microcalorimeter (MicroCal Inc., Northampton, MA, USA) with a 0.5 mL cell volume. The heat capacity vs. temperature profiles were obtained for a scanning rate of 1.0 °C min^−1^ in the 10–50 °C temperature range. A constant pressure of 1.8 atm was applied to the liquids in the cells. Calorimetric data were adjusted for the instrumental buffer–buffer baseline. At first, the buffer was placed in both the sample and reference compartments. A DSC curve corresponding to the buffer vs. buffer run was used as the instrumental baseline. The calorimetric data were corrected for the calorimetric baseline (by subtracting buffer–buffer scan). The DSC curves were analyzed with the use of MicroCal Origin Software.

#### 2.2.8. Statistical Analysis

All experiments and measurements in this study were carried out in triplicate and the data were expressed as a the mean + standard deviation (SD). The resulting data were analyzed using OriginPro 8.5.0 SR1 software (OriginLab Corporation, Northampton, MA, USA).

## 3. Results and Discussion

### 3.1. Encapsulation Degree Analysis

The analysis of the liposomal encapsulation degree of chloride-5-methyl-12(H)-quino[3,4-b]-1,4-benzothiazine (5-MBT) was performed spectrophotometrically for systems with a surrounding pH in the 4.47–6.10 range. The absorbance of 5-MBT was measured at 241 nm. The standard curves for the analyzed drug were obtained by plotting the absorbance against the concentration. Using these curves, the concentration of the non-encapsulated drug was determined in the supernatant. The encapsulation efficiency of the liposomes entrapping chloride-5-methyl-12(H)-quino[3,4-b]-1,4-benzothiazine was determined as the mass ratio between the amount of the drug incorporated in liposomes and the ratio used in the liposome preparation (see Equation (1)). The encapsulation efficiencies of 5-MBT in the liposomes are given in Table 1. Based on Equation (2), the drug loading (*DL*%) was determined and equaled to 6.29%, 6.30% and 6.28% for the pH values of 4.47, 5.59 and 6.10, respectively. The obtained results confirmed the influence of the buffer pH on the degree of the encapsulation of the tested compound.

### 3.2. Stability of Liposomal Preparations

**Figure 3:**
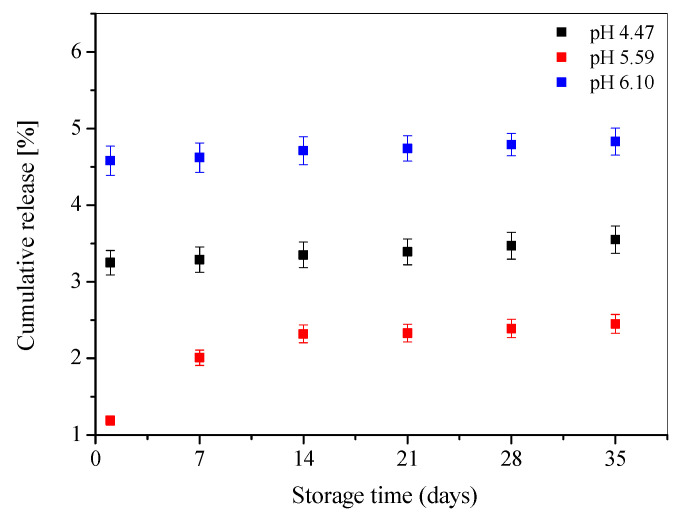
Drug release profiles of chloride-5-methyl-12(H)-chino[3,4-b]-1,4-benzothiazine (5-MBT) loaded in the liposomes obtained in different pH.

The research on novel potential carriers for therapeutic compounds is focused mainly on the assessment of their physical and chemical stability. These properties are the basis for determining the conditions of technological conduct, selection of auxiliary agents in the development of drug formulations and specification of their storage conditions. Physicochemical factors have a significant impact on both the durability of the medicinal substances themselves and their transport structures. The stability analysis of the obtained liposomal preparations was carried out over a period of 7 weeks. The samples were stored at +4 °C during the studied time period. The results of the stability studies, based on the weekly determination of the release rate, are shown in Figure 3. The R% analysis was performed based on Equations (4) and (5).

Several weeks of observing the liposomal preparations show that the greatest differences in the degree of drug release occurs in the case of liposomes prepared with buffer pH 5.59 whereas, for the liposomes in buffer pH 4.47 and 6.10, this process is more stabilized and the values of the compound permeation through the phospholipid membrane for these two cases oscillate within the limits of ±5% in the entire analyzed time range (Figure 3). It was also found that the process of 5-MBT release from liposomes in the period between the 2nd and 6th weeks of observation was almost constant. The obtained results show that the pH influences the stability of the tested L_DPPC/5-MBT_ liposomes, which is consistent with the results obtained with the EPR technique.

**Figure 4:**
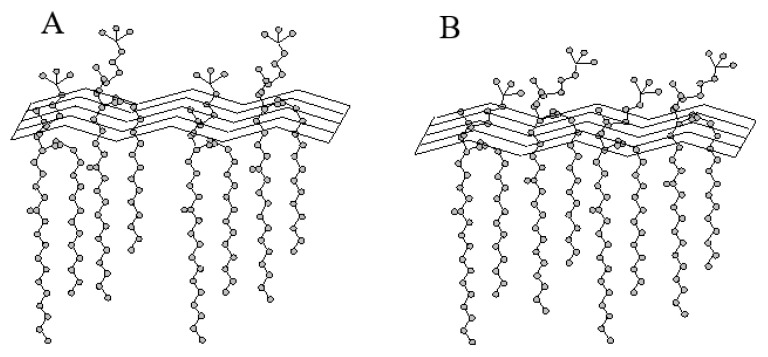
Schematic representation of the polar conformation of the phosphatidylcholine group. (**A**) Conformation with a perpendicular arrangement of the phospholipid heads to the membrane surface, and (**B**) conformation with a parallel arrangement of the phospholipid heads to the membrane surface.

The highest drug penetration through the phospholipid membranes can be observed for the liposomes in buffer having a pH of 6.10 (R% max~4.8%), whereas the lowest for liposomes with a buffer pH 5.59 (R% max~2.4%) (Figure 3). This is due to the conformation of the liposomal membrane and the arrangement of phospholipid “heads” in the phospholipid membrane in a given pH environment (Figure 4).

The obtained results correlate with the values obtained for the determined degree of 5-MBT encapsulation (Table 1). The lowest degree of encapsulation was obtained for pH 6.10 (70.37%), and the highest for pH 5.59 (83.08%).

The pH value undoubtedly plays a key role here. The deviation of the polar “heads” of phospholipids caused by the change in the pH of the buffer allows the 5-MBT to migrate to the surface of the membrane. In addition, the ionization of the phosphate groups may facilitate the movement of the compound molecules along the normal to the surface of the phospholipid membrane, which is associated with the disappearance of electrostatic interactions and hydrogen bonds formed by phosphate groups, and may cause conformational changes of the phosphate groups as well as the release of 5-MBT.

It is believed that a mobility gradient exists along the long axis of the phospholipid molecules in the bilayer. The least mobile segment of the phospholipid molecule is its glycerol skeleton, while the mobility increases towards the methyl ends of both acyl chains and towards the end of the polar head. The polar heads of membrane phospholipids are quite mobile. For example, in the case of phosphatidylethanolamines, their polar head takes a position, so that its axis is perpendicular to the fatty acid chains. This location allows for maximum intermolecular interactions [37].

Changes in the mobility of the polar heads of phospholipids account for the thermotropic changes observed on DSC grams. In phosphatidylethanolamine (PE), a 2.8 Å-long hydrogen bond is formed between the –NH_3_^+^ group of one molecule and the neighboring oxygen atom. In phosphatidylcholine PC, the –NH_3_^+^ group is replaced by a –N^+^(CH_3_)_3_ group, which does not form a hydrogen bond with the phosphate group of the adjacent molecule. As a consequence, the conformation of the PC’s polar head is different from that of PE’s [38]. The common feature of both phospholipids (PE and PC) is that the vector takes the distance between P–N atoms, a position at a small angle in relation to the bilayer plane.

In order to explain the process of releasing encapsulated compounds from liposomes, the analyzed problem should be considered at the molecular level. The fluidity of phospholipid membranes is, i.a., a reflection of molecular movements taking place in it. Molecular movements in membranes cover a very wide time range, from the molecular vibrations occurring in 10^−14^ s to the transversal movements of whole molecules, very slow, with a half-life on the order of days.

The movements occurring within one molecule (but decisive on the level of the entire membrane in terms of the release of liposome-encapsulated substance) include, first of all, rotations around single C–C bonds in the chain. These movements are not completely free. The smallest potential energy of a molecule occurs for three conformations: *anti-periplanar (t), gauche plus (g^+^) and gauche minus (g^−^)/synclinal/*.

The energy barrier of the *t–g* transition is approximately 3.5 kcal/mol. At room temperature, the thermal energy is high enough to cause rapid rotations from one conformation to another. In fatty acid chains, rotations can occur not only around a specific C–C bond, but can also spread up and down along the chain. Thus, it is highly probable that only those rotations will occur that result in an approximately linear configuration of the chain parallel to the adjacent one. It is possible that, for example, in the case of the simultaneous occurrence of two mirror rotations, *g^+^* and *g^−^*, they separated from each other by one C–C bond in the anti-periplanar configuration. The *g^+^tg^−^* and *g^−^g^+^* configurations, called *β*-conjugated, shorten the chain length by one –CH_2_ group, which is approximately 1.27 Å. This is called a minimal defect in volume that can slide along the chain and is believed to be the cause of bilayer permeability.

The preferred configuration of sat”rate’ fatty acid chains In bilayers is therefore a linear configuration, ensuring the most favorable interactions between adjacent chains (i.e., the one observed at 37 °C when the acyl chains are tightly packed and additionally arranged at an angle to the surface of the bilayer-phase *P_β’_*). Any structural (phase) changes occurring, e.g., as a result of temperature increase, will result in increased permeability.

In our study, the factor influencing the permeability of the L_DPPC/5-MBT_ liposome bilayer, apart from temperature was the environmental pH. The presence of hydrogen bonds between the DPPC molecules that make up the phospholipid bilayer results from the protonation of the phosphate group of phospholipids in an acidic environment.

**Figure 5:**
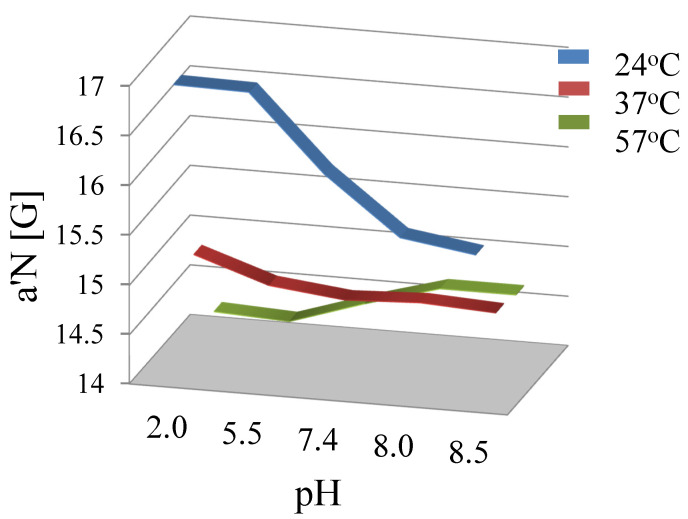
Effect of the pH and temperature on the polarity and the motion of spin marker 5-DOXYL along the perpendicular to the surface of membrane.

The results of the EPR tests and determination of the parameter *a*′*N*, point to the decreased ionization of phosphatidylcholine phosphate groups (Figure 5), accompanying the changes of the surrounding pH within the 2.0–8.5 value range and when the temperature (Tc 41 °C) remains below that for the main DPPC phase transition.

**Figure 6:**
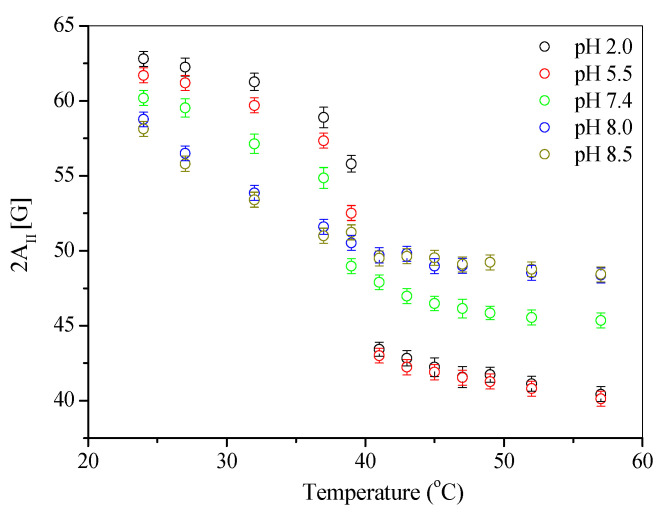
Effect of the temperature on maximum hyperfine splitting 2*A_II_* of 5-DOXYL in liposome vesicles.

The *a*′*N* parameter is typically used for describing changes in the polarity of liposomal membranes, depending on the polarity of the environment during liposomes’ preparation. An increase in the value of the *a*′*N* parameter indicates an increased polarity of the membrane [23]. The sigmoid curve, characterizing the changes in the *a*′*N* value also describes changes in the fluidity of phospholipid membranes. For the tested liposomes, the value of the *a*′*N* parameter, and thus the membrane polarity, decreases when the temperature is raised within the 24 °C to 37 °C range. It was also found that the *a*′*N* parameter values decreased with raised pH (2.0–8.5) at these temperatures. Above the phase transition temperature, Tc, changes of the parameter *a*′*N* values become inverse. Such changes are observed for liposomes prepared in both acidic and alkaline environments. This inversion can be explained by the movement of the 5-DOXYL marker perpendicular to the membrane surface along the aliphatic phospholipids chains. This movement may result from both the pH change and increased temperature; this is also indicated by the changes in the parameter 2*A_II_* (Figure 6).

The obtained results are consistent with the observed effect of DPPC liposomal membrane polarity on the distance increase between the spin marker and the membrane surface [23].

The pH change of the buffer used to prepare liposomes caused the movement of phospholipid polar heads, allowing for the transit of the label to the membrane surface. The ionization of the phosphate groups may facilitate shifting of the marker along the normal to the surface of the phospholipid membrane; this is associated with disappearance of electrostatic interactions and hydrogen bonds formed by phosphate groups and may cause conformational changes in the phosphate groups, which is also confirmed by the neutron spin-echo measurements [39].

The observed inversion of membrane stiffness (sudden change in the value of the 2*A_II_* parameter at a temperature of ca. 40 °C (Figure 6)) is also associated with the presence of hydrogen bonds, which stiffen the membranes at low temperatures (below Tc) in an acidic environment.

The mentioned hydrogen bond occurs between the phosphate group oxygen in one phospholipid molecule and the hydroxyl group hydrogen of the adjacent DPPC molecule. Apparently, when the temperature is raised, the *P_β’_*–*L_α_* phase transition is accompanied by the breaking of hydrogen bonds and the phospholipids in the bilayer are loosened. The reduction in the membrane stiffness with the increasing temperature is manifested in these systems by a decrease in the value of the 2*A_II_* parameter. The lowest value of 2*A_II_* is characteristic for the liposomes obtained in an acidic environment, compared to the systems in an alkaline environment (Figure 6).

### 3.3. In Vitro Drug Release Study

The amount of active ingredient released from a pharmaceutical preparation and dissolved in the surrounding body fluid as measured under laboratory conditions is known as pharmaceutical availability. This parameter is the same as the rate of the release process. It is very important because it determines the value characterizing the first stage in the system of drug transformation in the body (LADME). It is described as the percentage or fraction of the drug dose that is dissolved in the receiving fluid within a specified time. Only the free fraction of the therapeutic compound is absorbed and can exert its pharmacological effect. The structure of a drug (its form) must therefore be constructed so that the right amount of drug substance can be released from it in a timely manner. The rate and degree of release of the active ingredient depends primarily on the form of the drug. The excipients used and the method of preparing the drug also affect the release process. This fact became the basis for the search for new alternative forms of drug transport.

**Figure 7:**
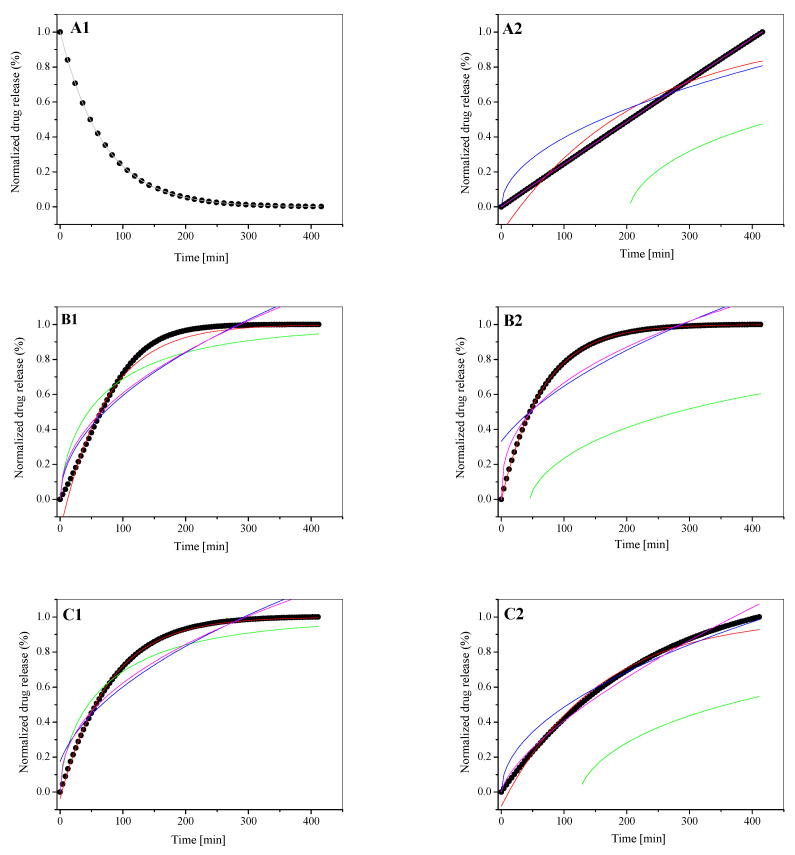
The release kinetics of 5-MBT from the L_DPPC/5-MBT_ liposomes (**A1**–**C1**) and their complexes with HSA [L_DPPC/5-MBT_]:HSA (**A2**–**C2**). (**A**) pH 4.47, (**B**) pH 5.59, (**C**) pH 6.10. Colored lines corresponding to the fits of the mathematical models. Black dot lines corresponding to the experimental data. (**-**) Korsmeyer–Peppas model; (**-**) first-order model; (**-**) Bhaskas model; (**-**) Higuchi model; (**-**) Ritger–Peppas model.

In order to analyze the process of the test compound released from the liposomes composed of dipalmitoylphosphatidylcholine as the main component of the membrane, five mathematical models were used (see Equations (6)–(10)) [20,27,30,31,32,33,34,35,36]. The release rate studies were carried out at 37 °C. Additionally, to maintain the physiological conditions, liposomes were treated with human serum albumin (HSA). The obtained [L_DPPC/5-MBT_]:HSA complex as well as the liposomal form of the test compound (L_DPPC/5-MBT_) were subjected to several hours of temperature incubation. The release profiles of the analyzed compound from the tested liposomes along with the matching of appropriate mathematical models are presented graphically (Figure 7). The use of mathematical models to describe the release process of the tested compound allowed for the determination of numerical values characterizing a given process (Table 2).

It was observed that the 5-MBT release profiles follow a similar pattern for L_DPPC/5-MBT_ liposomes obtained at buffer pH values of 5.59 and 6.10. However, they differed significantly from the system obtained at pH 4.47. The conformation of the membrane and, more precisely, the arrangement of the polar “heads” of phospholipids at pH 5.59 and 6.10, made the release of 5-MBT typical. Lowering the pH to 4.47 caused the formation of hydrogen bonds stiffening phospholipid molecules in their polar regions and thus limited the release process. The preferred conformation of the polar heads of phospholipids in the pH environment of 4.47 is most likely parallel to the plane of bilayers. Similar behavior, the stiffening of the phospholipid membrane with the above-mentioned hydrogen bonds, was observed for the liposomes at an environmental pH of ~2, analyzed by electron paramagnetic resonance [40]. Thus, the pH of the environment determines a specific conformation of phospholipids, which translates into the kinetics of the release process of the compound encapsulated in liposomes.

Usually, when liposomes are used for therapeutic or diagnostic purposes, the composition of the membrane is predominantly adjusted to show increased permeability to liposome-encapsulated substances at a pH between 5.00 and 6.30. Designing the so-called “pH-sensitive” liposomes makes use of the observation that pH in neoplastic tissues lies within this range [41,42,43].

The analysis of the 5-MBT release profiles for pH 5.59 and 6.10, both for its liposomal form and the resulting complex with HSA, shows that the typical character of the profile shape was retained. These processes, however, differ in their kinetics, which is reflected in the determined parameters (Table 2 and Table 3). They also differ from the release profile obtained at pH 4.47.

**Figure 8:**
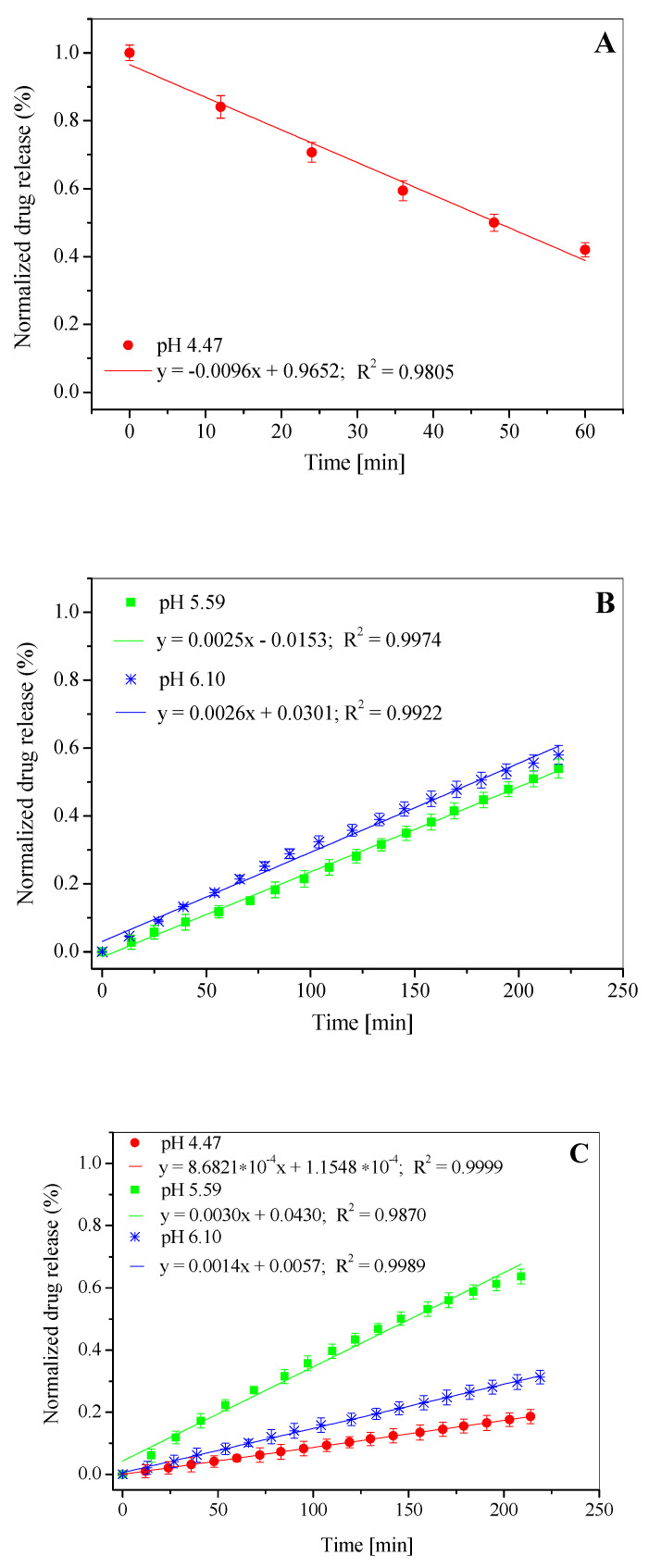
Release profile of 5-MBT from the L_DPPC/5-MBT_ liposomes (**A**,**B**) and them complexes with albumin [L_DPPC/5-MBT_]:HSA (**C**), at 37 °C, at initial time. Error bars represent standard deviation.

Among the analyzed group, [L_DPPC/5-MBT_]:HSA, the complex formed at pH 5.59 stands out (Figure 7(B2), Figure 8C). It shows the highest release kinetics and its profile differs from the systems obtained at pH 4.47 and 6.10 (Figure 7(A2–C2)).

A deeper comparative analysis of the release profiles with the time frame narrowed down to 3 h (Figure 8A–C), during which the release process is the fastest and most significant, demonstrates that the linear fit clearly indicates which pH value of the buffer used to prepare liposomes yields structures with the most desired release time. The obtained data show that the slope of y = ax + b for liposomes prepared in pH = 6.10 buffer is steeper than that for liposomes in pH = 5.59 buffer, making the compound release process faster. For liposomes prepared in pH = 4.47 buffer (Figure 7A), the release process of 5-MBT is significantly different. The 5-MBT percentage release was found to decrease proportionally with time. For these liposomes, a linear fit is possible only for the first hour of the analysis.

The values of *n* (Table 3) determined on the basis of Equation (9) indicate that, depending on the pH of the buffer, the release process either corresponds (or not) to the Fick’s diffusion.

As mentioned before, the value of *n* < 0.45 corresponds to the Fickian diffusion; 0.45 < *n* < 0.89 describes the non-Fickian process. Non-Fickian process means that the drug release takes place due to the both diffusion and controlled mechanisms [20]. The high value of the parameter *n* (0.985) obtained for the complex [L_DPPC/5-MBT_]:HSA, pH of 4.47, indicates the lack of direct participation of protein molecules in the release mechanism of the analyzed compound from phospholipid structures.

It was found that the maximum value of the released compound (plateau effect) was achieved for most of the analyzed systems at the third hour of temperature incubation, which is consistent with the data obtained for other tested compounds encapsulated in liposomes obtained with the mREV method [20,29]. The obtained results also refer to the liposomal complexes obtained for doxorubicin [44].

### 3.4. Analysis of the Thermotropic Changes in the Phospholipid Bilayers: A DSC Study

**Figure 9:**
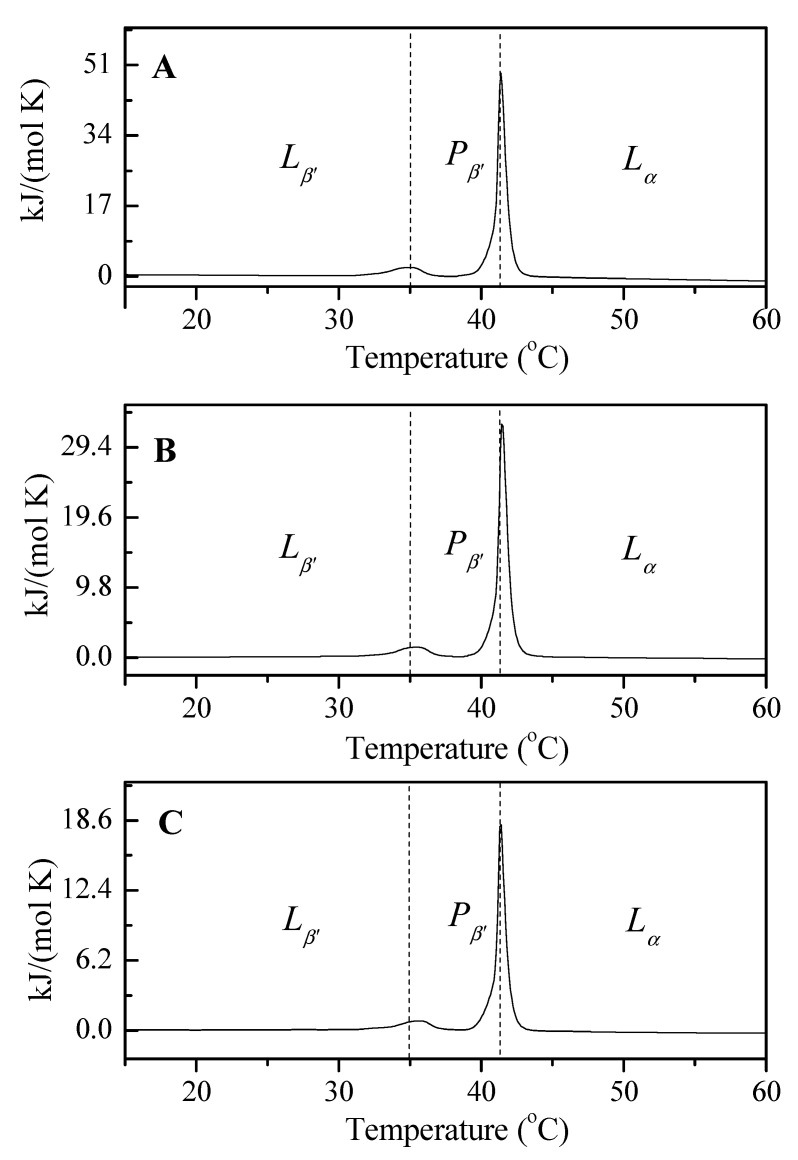
The influence of the pH environment on the temperature of L_DPPC/5-MBT_ liposomes phase transitions. (**A**) pH 4.47; (**B**) pH 5.59; (**C**) pH 6.10.

The DSC analysis of the thermotropic changes in liposome-forming phospholipids showed the effect of buffer pH and drug presence on both the phase transition temperature (Tp) and the main phase transition (Tc) temperature (Figure 9).

Our earlier studies on liposomes composed only of dipalmitoylphosphatidylcholine are consistent with the literature data on the temperature of DPPC phase transitions [45], therefore they can be compared to the results obtained for L_DPPC/5-MBT_ liposomes (Table 4). The determined temperature of the phase transition Tp from the tilted gel phase to the folded gel phase (*L_β’_–P_β’_*) for liposomes composed of only one membrane component, according to the literature, is 34.64 °C (Figure 9, dashed line), while the main phase transition temperature is 41.30 °C (Figure 9, dashed line). In the case of the tested liposomes containing 5-methyl-12(H)-quino[3,4-b] [1,4] benzothiazine chloride, it can be observed that the tested temperature Tp is higher than the above-mentioned value determined for the phospholipids forming model membranes devoid of the drug, which confirms the partial localization of the tested compound in the hydrophilic region of the membrane (Table 4). The main phase transition temperature did not change significantly compared to the Tc value of the reference liposomes (made from DPPC only). This means that the incorporation of 5-methyl-12(H)-quino[3,4-b] [1,4] benzothiazine chloride into the liposomes does not disturb the temperature of the main phase transition, which is of significant practical importance.

Each modification of the drug carrier formulation by adding another membrane component carries the risk of changes in the Tc temperature (e.g., its increase), or the emergence of additional phases that could affect the release process of the encapsulated compound. The Tc temperature of DPPC is comparable to the temperature of cancerous tissues, which ought to facilitate the release process. The conducted DSC studies indicate that the encapsulation of 5-MBT in liposomes does not significantly affect the temperature in question.

Analyzing the influence of the surrounding pH on the values of the thermodynamic potentials ΔH and ΔS determined by DSC (Table 4), a decrease in their values was found when the pH was raised. A detailed analysis of the phase transitions allows us to understand the organization of biological structures. Pre-transition (Tp) is less cooperative than Tc, and is also less energetic. This is the transition in which the tilted gel phase (*L_β’_*) change to ripple gel phase (*P_β’_*) occurs without increasing the hydration of the polar heads. In the (*P_β’_*) phase, the chains remain stretched and inclined, and the membrane surface is no longer flat, but exhibits ripples with rather high periodicity. Finally, the ripple gel phase *P_β’_* passes into the liquid crystal phase *L_α_*, in which the chains are not ordered but maintain the time-averaged positions perpendicular to the surface of the film. In the liquid crystal phase, lipids can rapidly diffuse in the bilayer plane, rotate, and undergo transitions from the *trans* to *gauche* conformation in the hydrocarbon chain part. The high cooperativity of this phase transition is characterized by a narrow peak on the DSC gram and high ΔH values, compared to those obtained for the pre-transition. The ΔH values determined for the liposomes obtained at pH 4.47 are more than twice the value of ΔH obtained for the liposomes obtained at pH 6.10.

## 4. Conclusions

Liposomes obtained by the mREV method have been well established as an effective drug delivery system, due to the simplicity of their preparation and unique characteristics. The research methods used in the analysis of the liposomal form of the compound with anticancer properties, 5-methyl-12(H)-quino[3,4-b] [1,4] benzothiazine chloride, allowed us to study the influence of temperature and pH on the process of 5-MBT release from L_DPPC/5-MBT_ liposomes and analyze the interaction of these liposomes with human serum albumin. The use of spectrophotometry and electron paramagnetic resonance recorded spectra provided data for the analysis of the emerging system/complex. Differential scanning calorimetry yielded information concerning the basic thermotropic parameters of the phospholipid membrane. On the basis of microcalorimetric analysis, it was found that 5-methyl-12(H)-quino[3,4-b] [1,4] benzothiazine chloride does not disturb the temperature of the main phase transition of phospholipids.

In order to evaluate the physicochemical properties of the L_DPPC/5-MBT_ liposomal structures, prospective carriers of 5-methyl-12(H)-quino[3,4-b] [1,4] benzothiazine chloride, the stability of the preparation and the degree of release of the encapsulated compound were analyzed. Our experiments were closely related to patient body temperature (37 °C). In addition, the observed data of drug release agree with the physicochemical properties of liposomes originated from DPPC.

The analysis of the physicochemical parameters indicates the actual possibility of obtaining a stable liposomal preparation containing 5-methyl-12(H)-quino[3,4-b] [1,4] benzothiazine chloride. The most optimal pH of the environment for the liposomal preparation of this compound is 5.59.

We believe that obtaining a stable liposomal preparation is a marked achievement, especially when it comes to novel compounds with prospective use as anti-cancer therapeutics. The obtained results confirm the possibility of using the mREV method for preparing a stable liposomal formulation of the novel compound with potential therapeutic usefulness.

## Figures and Tables

**Table 1:** Percentage of 5-MBT encapsulation in the liposomes obtained at different pH.

Liposome	Encapsulation Efficiency (*EE*%)		
	pH 4.47	pH 5.59	pH 6.10
L_DPPC/5-MBT_	75.76	83.08	70.37

**Table 2:** Fitting parameters of the 5-MBT release profiles from L_DPPC/5-MBT_ liposomes to different kinetic models.

L_DPPC/5-MBT_		Parameter		Korsmeyer–Peppas Eq	
4.47		SUM		1.321 × 10^−6^	
		k		1.001	
		t		0.985	
		n		0.45 < n < 0.89	
		R^2^_adj_		0.999	
**L_DPPC/5-MBT_**	**Parameter**	**First-Order Eq**	**Bhaskas Eq**	**Higuchi Eq**	**Ritger–Peppas Eq**
5.59	SUM	0.001	0.011	0.013	0.012
	α	11.871	−1.253 × 10^−24^	−1.727 × 10^−7^	−2.915 × 10^−44^
	k	0.013	0.012	0.059	0.069
	n	-	-	-	0.472
	R^2^_adj_	0.989	0.859	0.829	0.844
6.10	SUM	6.537 × 10^−5^	0.006	0.010	0.007
	α	2.741	−1.158 × 10^−25^	−9.175	−3.173 × 10^−42^
	k	0.013	0.012	0.057	0.084
	n	-	-	-	0.435
	R^2^_adj_	0.999	0.910	0.847	0.890

**Table 3:** Fitting parameters of the 5-MBT release profiles from the [L_DPPC/5-MBT_]:HSA complex to different kinetic models.

[L_DPPC/5-MBT_]:HSA	Parameter	First-Order Eq	Bhaskas Eq	Higuchi Eq	Ritger–Peppas Eq
4.47	SUM	0.004	0.202	0.013	8.522 × 10^−7^
	α	29.651	204.913	−6.737 × 10^−61^	−3.539 × 10^−9^
	k	0.004	0.002	0.039	0.002
	n	-	-	-	0.985
	R^2^_adj_	0.952	−0.673	0.846	0.999
5.59	SUM	1.776 × 10^−6^	0.257	0.01377	0.008
	α	0.195	45.700	−35.702	−1.513 × 10^−46^
	k	0.015	0.002	0.055	0.112
	n	-	-	-	0.386
	R^2^_adj_	0.999	−3.079	0.764	0.860
6.10	SUM	0.001	0.193	0.003	0.001
	α	11.907	125.132	−1.052 × 10^−32^	−2.574 × 10^−17^
	k	0.006	0.002	0.048	0.017
	n	-	-	-	0.686
	R^2^_adj_	0.987	−0.977	0.961	0.987

**Table 4:** Influence of buffer pH on the values of parameters ΔH, ΔS determined for the phospholipid pre-phase transition temperature (Tp) and the main phase transition (Tc) of phospholipids forming L_DPPC/5-MBT_ liposomes.

L_DPPC/5-MBT_		pH 4.47	pH 5.59	pH 6.10
*L_β’_*–*P_β’_*	T_p_ (°C)	34.84	35.35	35.56
	ΔS (kJ/mol∙K)	0.0233	0.0171	0.0097
	ΔH (kJ/mol)	7.1704	5.2780	3.0086
*P_β’_*–*L_α_*	T_c_ (°C)	41.37	41.43	41.39
	ΔS (kJ/mol∙K)	0.1441	0.0992	0.0539
	ΔH (kJ/mol)	45.335	31.211	16.939

## Data Availability

The data presented in this study are available on request from the corresponding author.
